# Tissue expression of lactate transporters (MCT1 and MCT4) and prognosis of malignant pleural mesothelioma (brief report)

**DOI:** 10.1186/s12967-020-02487-6

**Published:** 2020-09-04

**Authors:** Irene Dell’Anno, Elisa Barone, Luciano Mutti, Doris M. Rassl, Stefan J. Marciniak, Roberto Silvestri, Stefano Landi, Federica Gemignani

**Affiliations:** 1 Department of Biology, University of Pisa, Pisa, Toscana Italy; 2 Sbarro Institute for Cancer Research and Molecular Medicine, Temple University, Philadelphia, PA USA; 3 Royal Papworth Hospital NHS Trust, Papworth Road, Cambridge Biomedical Campus, Cambridge, B2 0AY UK; 4 Cambridge Institute for Medical Research, University of Cambridge, Cambridge, UK

**Keywords:** Malignant pleural mesothelioma (MPM), MCT1, MCT4, Basigin (CD147), Immunohistochemistry (IHC), Tissue microarray (TMA)

## Abstract

**Background:**

Malignant pleural mesothelioma (MPM) is an aggressive neoplasm of the pleura, mainly related to asbestos exposure. As in other solid tumors, malignant cells exhibit high glucose uptake and glycolytic rates with increased lactic acid efflux into the interstitial space. Lactate transport into and out of cells, crucial to maintaining intracellular pH homeostasis and glycolysis, is carried out by monocarboxylate transporters (MCTs) and the chaperone basigin (CD147). We set out to examine the clinical significance of basigin, MCT1 and MCT4 in the context of MPM and to evaluate their expression in relation to the evolution of the disease.

**Methods:**

We used immunohistochemistry to measure the expression of basigin, MCT1 and MCT4 in a cohort of 135 individuals with MPM compared to a series of 15 non-MPM pleura specimens. Moreover, by Kaplan–Meier and Cox analyses we evaluated whether an expression over the average of these markers could be associated with the patients’ overall survival (OS).

**Results:**

We detected positive staining of basigin, MCT1, and MCT4 in most MPM specimens. In particular, MCT4 was always positive in malignant tissues but undetectable in the 4 normal pleural specimens incorporated within the tissue microarray. This was confirmed in the additional series of 15 normal pleural samples. Moreover, MCT4 expression was significantly associated with reduced OS.

**Conclusion:**

In this study, the tissue expression of basigin did not prove to be exploitable as a diagnostic or prognostic marker for MPM patients. The expression of MCT1 was not informative either, being tightly correlated with that of basigin. However, the expression of MCT4 showed promise as a diagnostic/therapeutic and prognostic biomarker.

## Background

It is well-known that cancer cells can obtain energy by increasing their rates of glycolysis despite oxygen being available (Warburg effect) [1]. This metabolic shift generates intracellular lactic acid that is actively expelled causing extracellular acidification. Low interstitial pH has been associated with increased levels of VEGF, IL8, and heparanase that could trigger cancer autocrine stimulation and enhance tumor aggressiveness [2]. Indeed, the accumulation of lactate in a wider range of solid cancers has been correlated with poor clinical outcome [3]. The monocarboxylate transporters (MCTs) are important players in this phenomenon. They mediate the co-transport of lactate with a proton, thus regulating intracellular and interstitial pH to maintain high glycolytic rates. MCT1 and MCT4 (encoded by the *SLC16A1* and *SLC16A3* genes, respectively) are considered the most important transporters in this process. Their activities appear to differ somewhat, with MCT1 mainly involved in the influx of lactate, whereas MCT4 is more important in the efflux of lactate [4]. A third player is basigin, a type I transmembrane glycoprotein (also known as CD147) and encoded by the *BSG* gene [5]. Basigin functions as a chaperone protein, by interacting with MCTs and stabilizing their localization at the cell membrane. Deregulated expression of basigin, MCT1, and MCT4 has been reported in carcinoma of pancreas [6], uterine cervix [7], kidney (papillary and clear renal cell) [8] and bladder urothelium [9], suggesting that these molecules could represent attractive targets for novel therapies. Malignant pleural mesothelioma (MPM) frequently contains highly hypoxic regions that might contribute to its aggressive behavior through the activation of the transcription factor HIF-1α [10]. However, MCT transporters and basigin have not been thoroughly investigated in MPM. Thus, it is conceivable that the expression of basigin, MCT1 and MCT4 could be related to MPM carcinogenesis and aggressiveness. To test this hypothesis, we assessed whether the expression of these molecules (evaluated by immunohistochemical staining, IHC) was associated with the malignant state or patients’ prognosis. The IHC analysis was performed on a tissue microarray (TMA) composed of diagnostic tissues from 135 individuals with MPM [11] and four samples of normal pleura. Fifteen additional samples from normal pleura were also employed for a further validation of the results. The prognostic analysis was carried out with univariate and multivariate statistical tests where the histological subtype of mesothelioma (epithelioid, sarcomatoid, or biphasic) [12] was included into the model. To date, histological subtype remains the main prognostic parameter for MPM patients [13, 14], with the epithelioid subtype having a better outcome than the sarcomatoid or biphasic MPM.

## Materials and methods

### Ethical considerations

This study was approved via the generic Royal Papworth Hospital Tissue Bank ethical approval granted by the Cambridge Research Ethics Committee (REC reference 08/H0304/56+5). The TMA sections used in this study were obtained from a TMA produced for a previously approved study (REC reference 09/H0311/21) [11]. For inclusion in the TMA, patients diagnosed with malignant mesothelioma between 2005 and 2010 had been previously identified from hospital records. Their histology slides and FFPE (formalin-fixed-paraffin-embedded) biopsy tissue blocks were retrieved from the pathology archive and clinical data relating to each case were extracted from hospital records.

### MPM tissue microarray and immunohistochemistry

The MPM TMA slides employed in this study were derived from the MPM TMA blocks previously constructed and used in the study by Dalton et al. [11]. Tissue cores of samples from 135 patients diagnosed with malignant mesothelioma were included in the study. Immunohistochemistry was performed as described in the study by Dalton et al. [11]. We employed the following antibodies: basigin (dilution 1:250; AB49493 Abcam), MCT1 (dilution 1:400; AB3538P Merk Millipore), or MCT4 (dilution 1:200; 376101 (F-10), Santa Cruz).

### Scoring and statistical analysis

Scoring was performed, using PathXL software, by two independent and experienced histopathologists. The slides were scored semi-quantitatively and an average of staining intensity at the nuclear, cytoplasmic and membrane level was obtained for each patient. The sum of these scores provided the patients’ total value of intensity for basigin, MCT1, and MCT4 expression. Among positive patients, the average was calculated and employed as a cut-off value to define “low expressing” or “high expressing” patients. The use of the average intensity allowed a more conservative definition of the “high expressing patients” as compared to the median being more sensitive to high values in skewed distributions [15, 16].

Statistical comparisons were performed using log-rank tests for censored data. Statistical analyses were performed using R and GraphPad software. The Kaplan–Meier method was used to analyse the overall survival (OS). Correlation between variables was determined using the Spearman Rank test. Survival was calculated from the last day of treatment. Univariate log-rank test was used to calculate the significance between the survival curves. The Cox proportional hazards regression model was performed to estimate the independence of factors which correlated in univariate analysis with survival. Hazard ratios (HRs) were presented with 95% confidence intervals (95% CI). A P value of less than 0.05 was considered statistically significant.

### Evaluation of basigin, MCT1, and MCT4 expression in normal pleura samples

The antibodies and protocols used for the staining of normal pleura samples were the same as those used for the TMA IHC. Fifteen normal pleura samples were stained together with some selected cases of MPM, previously studied in the TMA IHC, in order to compare the intensity and the localization of staining. The cases included lung tissue with some surface visceral pleural mesothelial cells and strips of parietal pleura from pneumothorax patients.

## Results

**Fig. 1:**
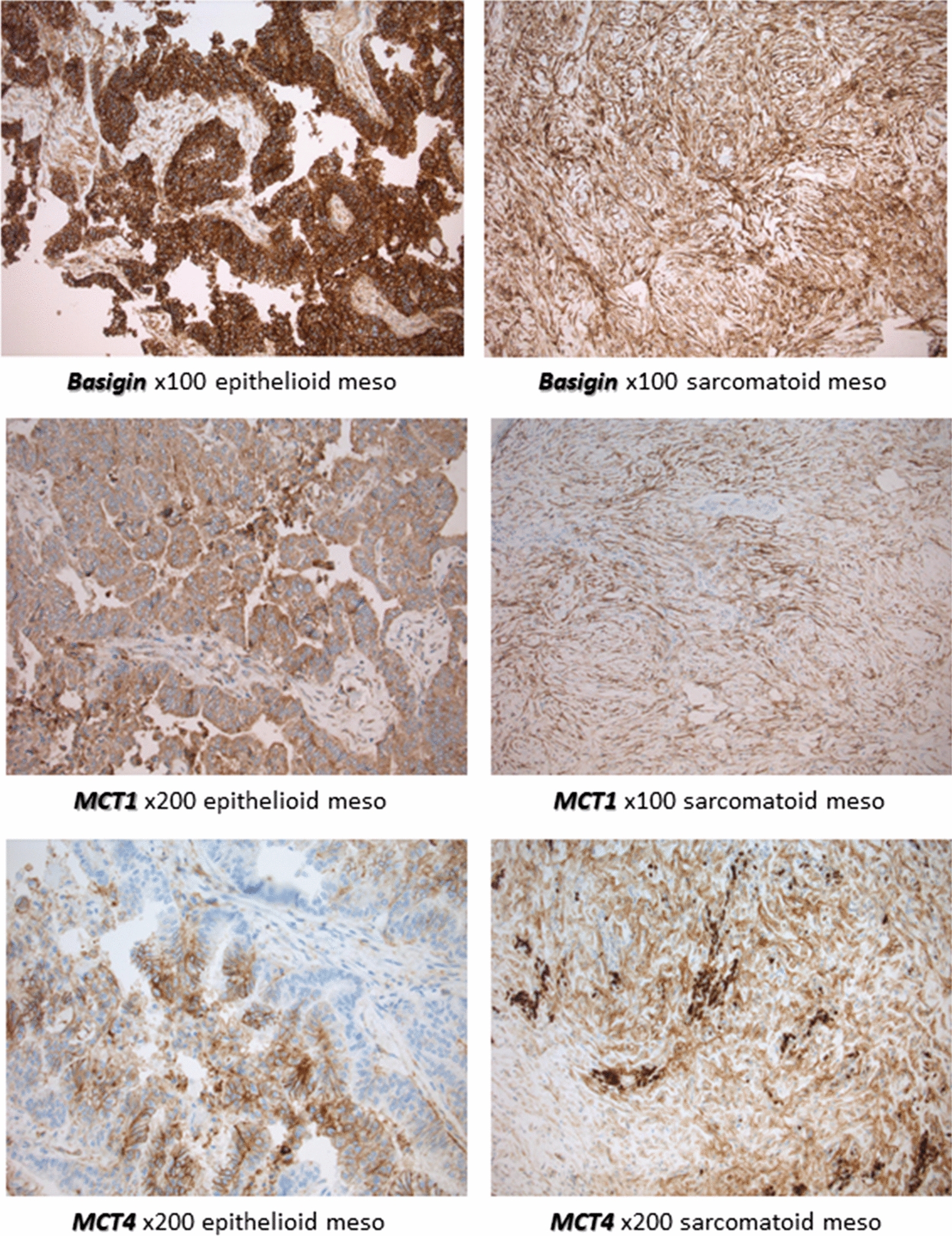
Immunohistochemistry demonstrating staining patterns for glycolytic markers, basigin, MCT1 and MCT4, in malignant pleural mesothelioma (MPM) cases. Representative images of epithelioid and sarcomatoid subtypes of mesothelioma showing membranous and cytoplasmic staining—basigin (top), MCT1 (middle), and MCT4 (bottom) staining

In order to evaluate further the expression of basigin, MCT1, and MCT4 in MPM, an IHC evaluation was performed on a TMA containing 135 samples. Expression scores were obtained from 89, 88 and 90 patients, respectively. All markers analyzed exhibited cytoplasmic and/or plasma membrane staining, with a predominance of the latter, as expected for membrane pumps. These metabolism-related proteins were strongly expressed in both epithelioid and sarcomatoid MPM (Fig. 1). Interestingly, we observed a co-expression of MCT1, but not MCT4, with basigin (P < 0.0001; Additional file 1: Figure S1 and Additional file 2: Table S1).

**Fig. 2:**
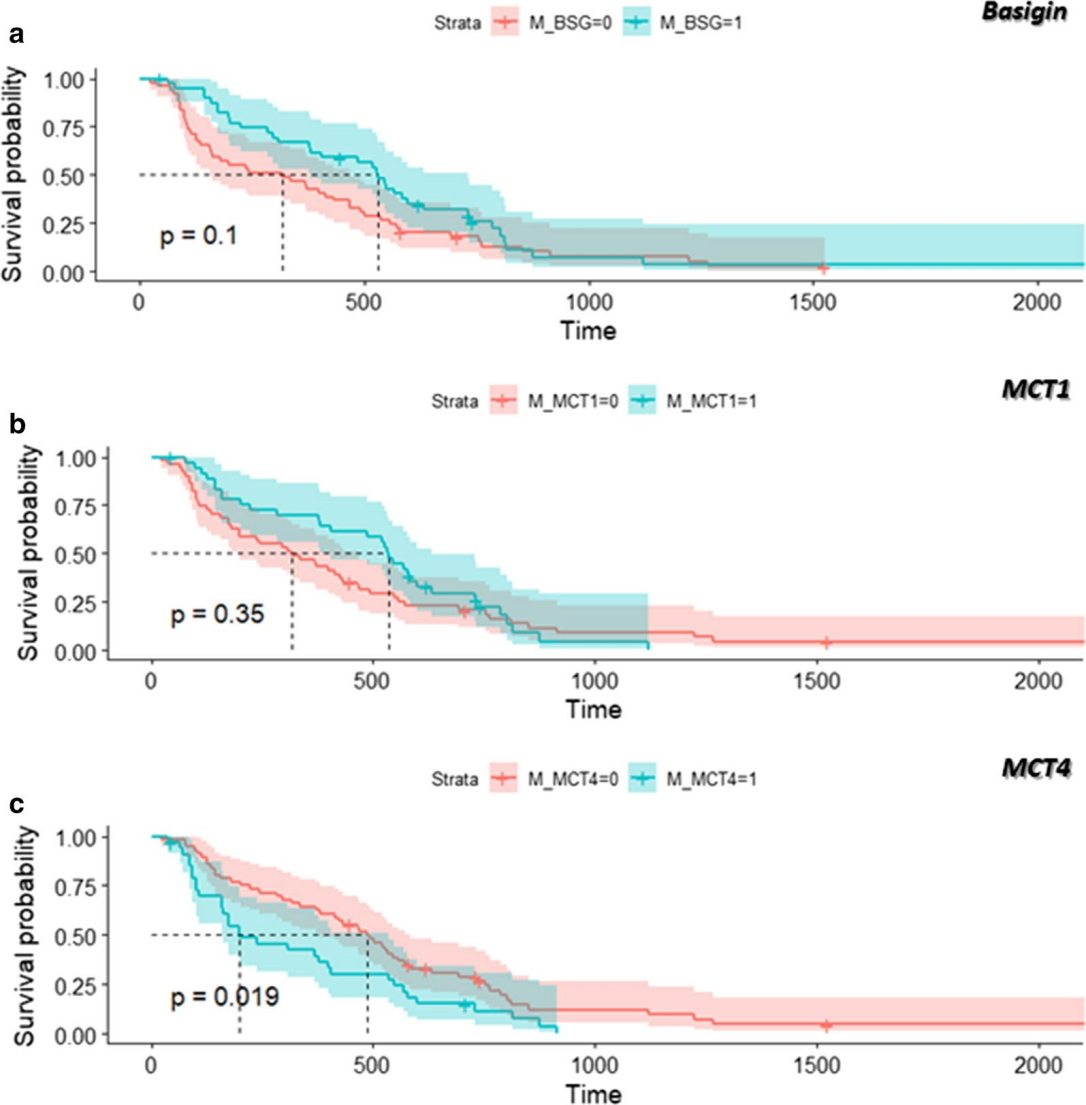
Kaplan–Meier analysis of the association of **a** basigin (low expression, n = 49; high expression, n = 40), **b** MCT1 (low expression, n = 51; high expression, n = 37), and **c** MCT4 (low expression, n = 56; high expression, n = 34) expression with overall survival (OS) among 88–90 patients. Blue line, patients showing high (H) marker expression; red line, patients showing low (L) marker expression

The expression of each marker was classified as low or high and analyzed in relation to the OS by the Kaplan–Meier method. High expression of MCT4 was significantly associated with shortened OS (log-rank P = 0.019, HR = 1.70; 95% CI 1.09–2.70). Patients with low expression of total MCT4 survived longer than patients with high expression. At one, two or three years after diagnosis, the OS of patients with low MCT4 was 70%, 40% and 20% respectively, as compared to 40%, 20% and 0% for patients with high MCT4 (Fig. 2). When only patients with epithelioid MPM were analysed (n = 68), the association between the OS and MCT4 was less evident (log-rank P = 0.053, HR = 1.69, 95% CI 0.99–2.88) likely due to reduced statistical power. The same trend was observed for the other histological subtypes (n = 22 overall) lacking any statistical significance due to the low power of the analyses. The Kaplan–Meier analyses, stratified by histological subtype, are provided in supplementary materials (Additional file 3: Figure S2). Similar results for MCT4 were obtained also in a multivariate analysis which included age, gender, tumor histological subtype, previous cancer diagnosis, type of therapy (surgery, radio- or chemotherapy), and mesothelial marker staining (WT1 and calretinin). In this analysis, low expression of calretinin and lack of therapeutic intervention were associated with a statistically significant short OS (Table 1). The results of the univariate analyses for each of the considered factors are reported in Table 2 and supplementary materials (Additional file 4: Figure S3). Overall, we could not find any statistically significant association between MPM patients’ OS and basigin or MCT1 levels (Fig. 2).

**Table 1:** Worse prognostic factors affecting OS by multivariate analyses

Variables	Unfavorable factor	HR (95% CI)	P
MCT4	High expression	1.67 (0.99–2.81)	0.054
Calretinin	Low expression	0.25 (0.098–0.65)	0.004**
Therapeutic intervention	Chemo/radio therapy or surgery	0.40 (0.17–0.92)	0.030*

Statistical significance is indicated by asterisk (*), where * = P < 0.05, ** = P < 0.01, *** = P < 0.001

**Table 2:** Univariate analysis of clinicopathologic variables (calretinin expression, WT1 expression, histological subtype, therapy intervention, gender and previous cancer diagnosis) for overall survival in MPM patients, expressed as 95% confidence interval (95% Cl) and P-value (log-rank test)

Variables	HR (95% CI)	P
Calretinin	0.24 (0.14–0.42)	< 0.0001***
WT1	0.52 (0.27–1.03)	0.06
Histotype	2.28 (1.51–3.44)	< 0.0001***
Therapeutic intervention	0.47 (0.27–0.82)	0.006**
Gender	0.73 (0.46–1.17)	0.19
Previous cancer	0.88 (0.50–1.54)	0.65

Statistical significance is indicated by asterisk (*), where * = P < 0.05, ** = P < 0.01, *** = P < 0.001

In order to extend these observations, we queried the TCGA online resource (https://portal.gdc.cancer.gov/). In this database, transcriptomic information is available for 87 MPM samples. We extracted the expression levels of *SLC16A3* (MCT4) and applied the Kaplan–Meyer method for analyzing the OS. This analysis showed that a low mRNA expression of *SLC16A3* is associated with longer OS, consistent with our findings at the protein level. The median survival for patients with a high *SLC16A3* expression was 400 days, but this value doubled in those patients with low *SLC16A3* expression (Additional file 5: Figure S4; log-rank P = 0.004, HR = 2.47, 95% CI 1.34–4.53).

**Fig. 3:**
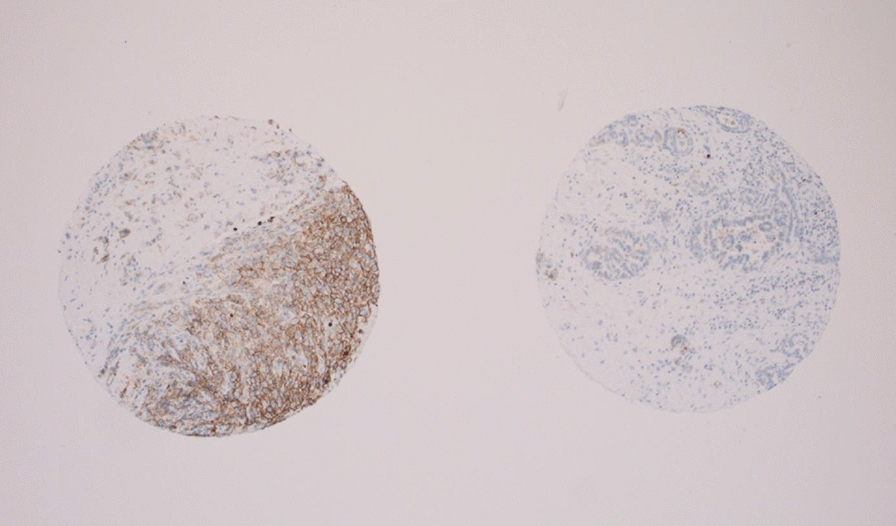
TMA after application of MCT4 antibody. A representative view (magnification X50) of an individual mesothelioma case positive for MCT4, on the left, and a non-malignant pleura tissue negative for MCT4 protein, on the right

We next examined the expression of MCT4 in normal pleura. Sections of non-neoplastic pleura were obtained from specimens of fifteen individuals who had undergone bullectomy and pleurectomy for recurrent pneumothorax, and who had previously given research tissue bank consent. These sections were stained together with selected cases of MPM in order to compare the intensity and localization of the signals. Interestingly, all samples of non-neoplastic mesothelial tissue were negative for MCT4 (Fig. 3 and Additional file 6: Figure S5), but positive for basigin and MCT1 (Additional file 7: Figure S6).

## Discussion

In the present work, 135 core biopsy samples of MPM were examined by IHC for the expression of basigin, MCT1 and MCT4. Interestingly, we observed positive and cell compartment-specific staining for the three markers and a statistically significant co-expression of MCT1 and basigin in MPM tissues. This latter result is in agreement with previous studies reporting a positive association between MCT1, but not MCT4, and basigin expression in breast cancer and adrenocortical carcinomas [17, 18]. However, basigin or MCT1 levels were not found to be associated with the considered clinical variables. On the other hand, a high expression of MTC4 was found to be significantly associated with a short OS of MPM patients and a similar trend (at mRNA level) was also confirmed by the data available within the TCGA data portal. In addition, a lack of expression of MCT4 was noticed for the 4 samples of healthy pleura incorporated within the TMA, suggesting a sharp difference between benign and malignant tissues. Thus, we verified this finding by extending the IHC analysis on 15 normal pleura tissues. It is noteworthy that non-malignant tissues were negative for the expression of MCT4. This is in agreement with a previous observation reporting that the IHC expression of MCT4 was poor or lacking in a series of 20 samples of reactive mesothelial hyperplasia and markedly increased in a series of 35 human MPM [19]. Taken together these data strongly suggest that MCT4 is a potential prognostic bio-marker for human MPM. However, we are aware that, in the view of an application in a defined clinical context, the determination of the sensitivity and predictive value must be addressed in further studies [13, 20].

Increased expression of MCT4 by cancer cells enhances the interstitial concentrations of lactic acid and is, at least in part, responsible for the acidic tumour microenvironment. Acidosis promotes extracellular matrix degradation and angiogenesis, inhibits the immune response and it is toxic for non-transformed cells [21]. Acidosis can also favour cell motility and invasion and could enhance the resistance to glucose-deprivation-induced apoptosis that could be reverted by buffering the interstitial pH towards physiological values [22]. The increased expression of MCT4 in MPM tissues is in agreement with previous observations for other solid cancers. Elevated MCT4 levels have been suggested as biomarker of poor prognosis in breast, bladder, pancreatic, hepatocellular, gastric, oral squamous cell and clear cell renal carcinoma, melanoma, soft tissue sarcomas, osteosarcoma, and lung adenocarcinoma (an extensive literature reference can be found in the Additional file 8) . MPM can now be added to this list. Furthermore, the association between high expression of MCT4 and decreased survival has also been documented for colorectal cancer with peritoneal carcinomatosis. High MCT4 expression has been shown to correlate with enhanced metastatic processes, especially in oesophageal adenocarcinoma [23] and in colorectal carcinoma [24, 25], and with invasion in gastric cancer [26].

## Conclusions

In summary, we propose that MCT4 tissue expression could serve as a novel prognostic biomarker for patients with MPM and, given its biological role in cancer progression, a target for novel inhibitors in the fight of cancer [27]. Furthermore, it is conceivable that the inhibition of MCT4 and the consequent reduced interstitial acidosis could enhance the response to immune checkpoint inhibitors [21]. Finally, the collection of a broader number of healthy and malignant specimens could help to confirm whether MCT4 could also be exploited as a diagnostic biomarker.

## Supplementary information

**Additional file 1: Figure S1.** Scatter plot representing the correlation between MCT1 and basigin IHC staining.

**Additional file 2: Table S1.** Correlation between IHC staining of BSG, MCT1, and MCT4, expressed as Pearson’s correlation coefficient r, and 95% confidence interval (95%Cl), r^2^ and P-value of the simple regression model. Statistical significance is indicated by asterisks (*), where * = P < 0.05; ** = P < 0.01; *** = P < 0.001, and **** = P < 0.0001.

**Additional file 3: Figure S2.** Kaplan-Meier analysis of the association of basigin, MCT1, and MCT4 expression with overall survival (OS) among 87–90 patients, stratified by histological subtype (“**E**” epithelioid or “**O**” other, including sarcomatoid and biphasic). Blue line: patients showing high marker expression; red line: patients showing low marker expression.

**Additional file 4: Figure S3.** Association of OS with calretinin expression (0, red line = no relevance; 1, blue line = present), WT1 expression (0, red line = no relevance; 1, blue line = present), histological subtype (1, red line = epithelioid; 2, blue line = sarcomatoid, spindle and biphasic), therapy intervention (0, red line = no therapy; 1, blue line = any kind of therapy), gender (1, red line = male, 2, blue line = female) and previous cancer diagnosis (0, red line = no; 1, blue line = yes).

**Additional file 5: Figure S4.** Kaplan-Meier analysis of the association of *SLC16A3* (encoding for MCT4) expression with OS for patients recorded in the Mesothelioma TCGA database (“TCGA-MESO”). Blue line, patients showing high (H) marker expression; red line, patients showing low (L) marker expression.

**Additional file 6: Figure S5. MCT4 staining in normal pleura samples.** Seven representative pictures of fifteen normal pleura samples (acquired at 200X) showing that the staining with the MCT4 antibody in normal mesothelial cells provides largely negative results.

**Additional file 7: Figure S6. Basigin and MCT1 staining in normal pleura samples.** Representative pictures of normal pleura samples showing that the staining with **(A)** basigin (acquired at 200X) or **(B)** MCT1 antibody (acquired at 200X) in normal mesothelial cells provides largely positive results.

**Additional file 8: Additional literature references.** Extensive literature references reporting MCT4 as putative biomarker in human cancers.

## Data Availability

The data that support the findings of this study are available from the corresponding author upon reasonable request.

## Abbreviations

IHC: immunohistochemistry
MCT: monocarboxylate transporter
MPM: malignant pleural mesothelioma
OS: overall survival
TMA: tissue microarray

## References

[1] Cairns RA, Harris IS, Mak TW (2011). Regulation of cancer cell metabolism. Nat Rev Cancer.

[2] Peppicelli S, Bianchini F, Calorini L (2014). Extracellular acidity, a "reappreciated" trait of tumor environment driving malignancy: perspectives in diagnosis and therapy. Cancer Metastasis Rev.

[3] Kennedy KM, Dewhirst MW (2010). Tumor metabolism of lactate: the influence and therapeutic potential for MCT and CD147 regulation. Future Oncol.

[4] Draoui N, Feron O (2011). Lactate shuttles at a glance: from physiological paradigms to anti-cancer treatments. Dis Models Mech.

[5] Le Floch R, Chiche J, Marchiq I, Naiken T, Ilc K, Murray CM (2011). CD147 subunit of lactate/H+ symporters MCT1 and hypoxia-inducible MCT4 is critical for energetics and growth of glycolytic tumors. Proc Natl Acad Sci USA.

[6] Schneiderhan W, Scheler M, Holzmann K-H, Marx M, Gschwend JE, Bucholz M (2009). CD147 silencing inhibits lactate transport and reduces malignant potential of pancreatic cancer cells in in vivo and in vitro models. Gut.

[7] Pinheiro C, Longatto-Filho A, Pereira SMM, Etlinger D, Moreira MAR, Jubé LF (2009). Monocarboxylate transporters 1 and 4 are associated with CD147 in cervical carcinoma. Dis Markers.

[8] Kim Y, Choi J-W, Lee J-H, Kim Y-S (2015). Expression of lactate/H^+^ symporters MCT1 and MCT4 and their chaperone CD147 predicts tumor progression in clear cell renal cell carcinoma: immunohistochemical and The Cancer Genome Atlas data analyses. Hum Pathol.

[9] Choi JW, Kim Y, Lee J-H, Kim Y-S (2014). Prognostic significance of lactate/proton symporters MCT1, MCT4, and their chaperone CD147 expressions in urothelial carcinoma of the bladder. Urology.

[10] Nabavi N, Bennewith KL, Churg A, Wang Y, Collins CC, Mutti L (2016). Switching off malignant mesothelioma: exploiting the hypoxic microenvironment. Genes Cancer.

[11] Dalton LE, Clarke HJ, Knight J, Lawson MH, Wason J, Lomas DA (2013). The endoplasmic reticulum stress marker CHOP predicts survival in malignant mesothelioma. Br J Cancer.

[12] Fels Elliott DR, Jones KD (2020). Diagnosis of mesothelioma. Surg Pathol Clin.

[13] Ascoli V, Murer B, Nottegar A, Luchini C, Carella R, Calabrese F (2018). What’s new in mesothelioma. Pathologica.

[14] Ceresoli GL, Locati LD, Ferreri AJ, Cozzarini C, Passoni P, Melloni G (2001). Therapeutic outcome according to histologic subtype in 121 patients with malignant pleural mesothelioma. Lung Cancer.

[15] Whitley E, Ball J (2002). Statistics review 1: presenting and summarising data. Crit Care.

[16] Mishra P, Pandey CM, Singh U, Keshri A, Sabaretnam M (2019). Selection of appropriate statistical methods for data analysis. Ann Card Anaesth.

[17] Pinheiro C, Sousa B, Albergaria A, Paredes J, Dufloth R, Vieira D (2011). GLUT1 and CAIX expression profiles in breast cancer correlate with adverse prognostic factors and MCT1 overexpression. Histol Histopathol.

[18] Pinheiro C, Granja S, Longatto-Filho A, Faria AM, Fragoso MC, Lovisolo SM (2015). Metabolic reprogramming: a new relevant pathway in adult adrenocortical tumors. Oncotarget.

[19] Mogi A, Koga K, Aoki M, Hamasaki M, Uesugi N, Iwasaki A (2013). Expression and role of GLUT-1, MCT-1, and MCT-4 in malignant pleural mesothelioma. Virchows Arch.

[20] Simon R (2015). Sensitivity, specificity, PPV, and NPV for predictive biomarkers. J Natl Cancer Inst..

[21] Dhup S, Dadhich RK, Porporato PE, Sonveaux P (2012). Multiple biological activities of lactic acid in cancer: influences on tumor growth, angiogenesis and metastasis. Curr Pharm Des.

[22] Chiche J, Brahimi-Horn MC, Pouysségur J (2010). Tumour hypoxia induces a metabolic shift causing acidosis: a common feature in cancer. J Cell Mol Med.

[23] Huhta H, Helminen O, Palomäki S, Kauppila JH, Saarnio J, Lehenkari PP (2017). Intratumoral lactate metabolism in Barrett's esophagus and adenocarcinoma. Oncotarget.

[24] Nakayama Y, Torigoe T, Inoue Y, Minagawa N, Izumi H, Kohno K (2012). Prognostic significance of monocarboxylate transporter 4 expression in patients with colorectal cancer. Exp Ther Med.

[25] Martins SF, Amorim R, Viana-Pereira M, Pinheiro C, Costa RF, Silva P (2016). Significance of glycolytic metabolism-related protein expression in colorectal cancer, lymph node and hepatic metastasis. BMC Cancer.

[26] Lee JY, Lee I, Chang WJ, Ahn SM, Lim SH, Kim HS (2016). MCT4 as a potential therapeutic target for metastatic gastric cancer with peritoneal carcinomatosis. Oncotarget.

[27] Futagi Y, Kobayashi M, Narumi K, Furugen A, Iseki K (2018). Identification of a selective inhibitor of human monocarboxylate transporter 4. Biochem Biophys Res Commun.

