# Supplementary figures and images for: Tissue expression of lactate transporters (MCT1 and MCT4) and prognosis of malignant pleural mesothelioma (brief report)
Source: J Transl Med. 2020 Sep 4;18:341. doi: 10.1186/s12967-020-02487-6 (PMC7650278; doi:10.1186/s12967-020-02487-6)

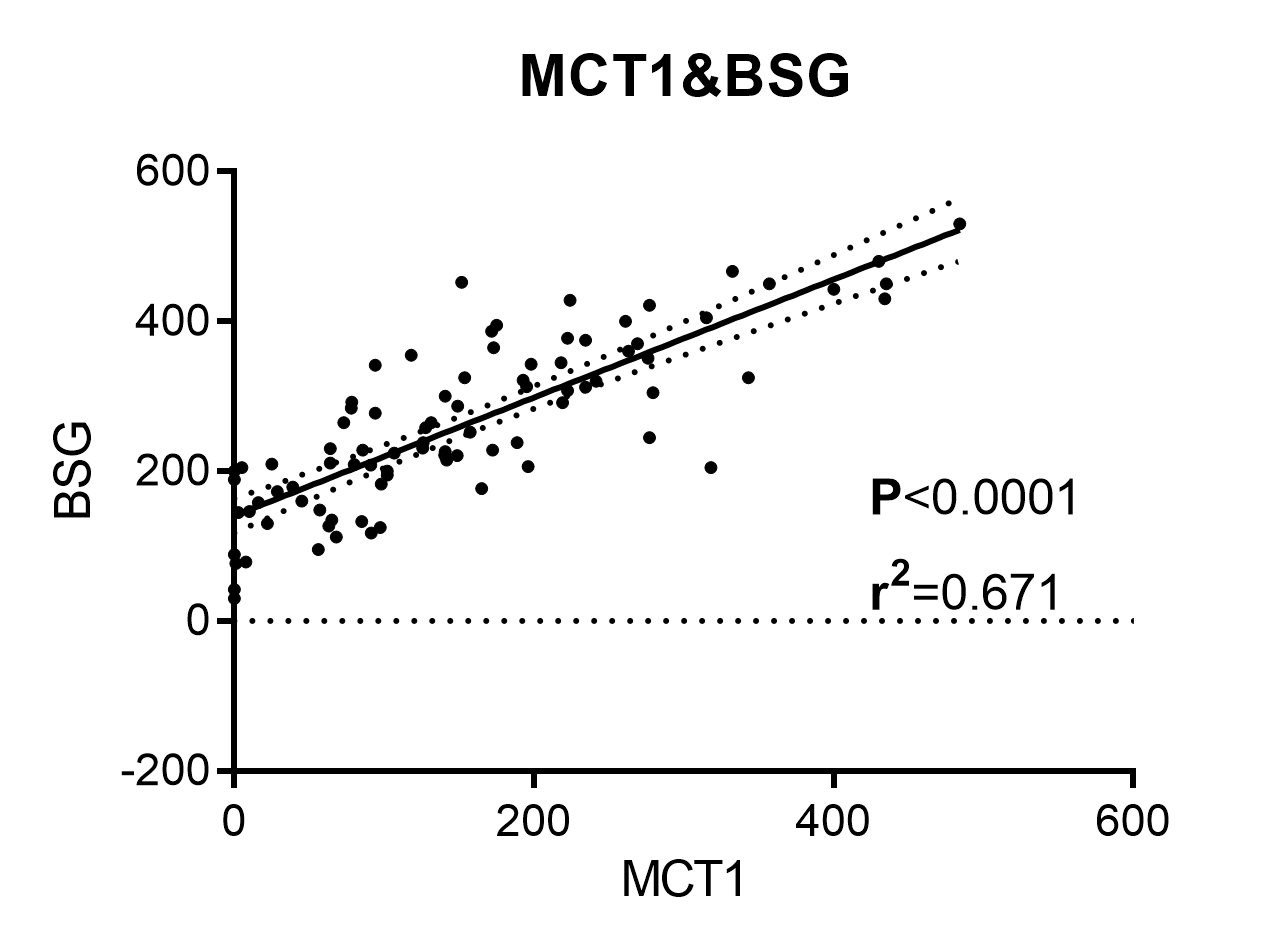

Supplement: Supplementary file 1 — Additional file 1: Figure S1. Scatter plot representing the correlation between MCT1 and basigin IHC staining. [file 12967_2020_2487_MOESM1_ESM.tif]

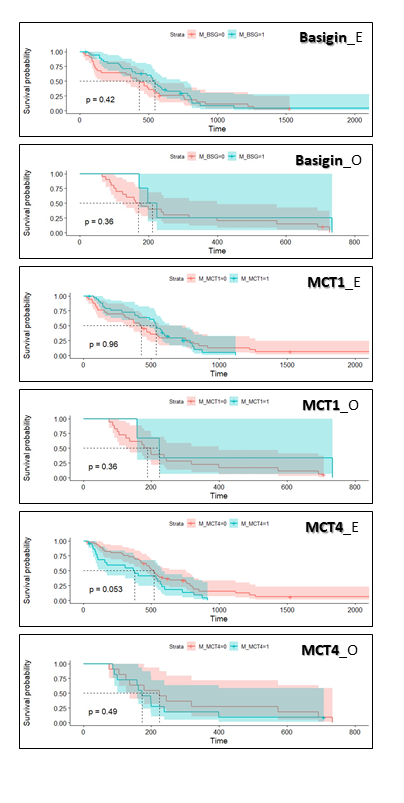

Supplement: Supplementary file 3 — Additional file 3: Figure S2. Kaplan-Meier analysis of the association of basigin, MCT1, and MCT4 expression with overall survival (OS) among 87–90 patients, stratified by histological subtype (“E” epithelioid or “O” other, including sarcomatoid and biphasic). Blue line: patients showing high marker expression; red line: patients showing low marker expression. [file 12967_2020_2487_MOESM3_ESM.tif]

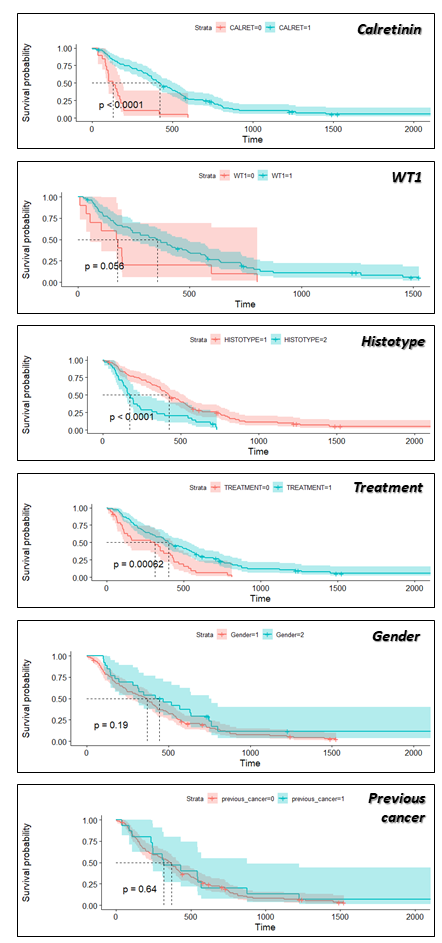

Supplement: Supplementary file 4 — Additional file 4: Figure S3. Association of OS with calretinin expression (0, red line = no relevance; 1, blue line = present), WT1 expression (0, red line = no relevance; 1, blue line = present), histological subtype (1, red line = epithelioid; 2, blue line = sarcomatoid, spindle and biphasic), therapy intervention (0, red line = no therapy; 1, blue line = any kind of therapy), gender (1, red line = male, 2, blue line = female) and previous cancer diagnosis (0, red line = no; 1, blue line = yes). [file 12967_2020_2487_MOESM4_ESM.tif]

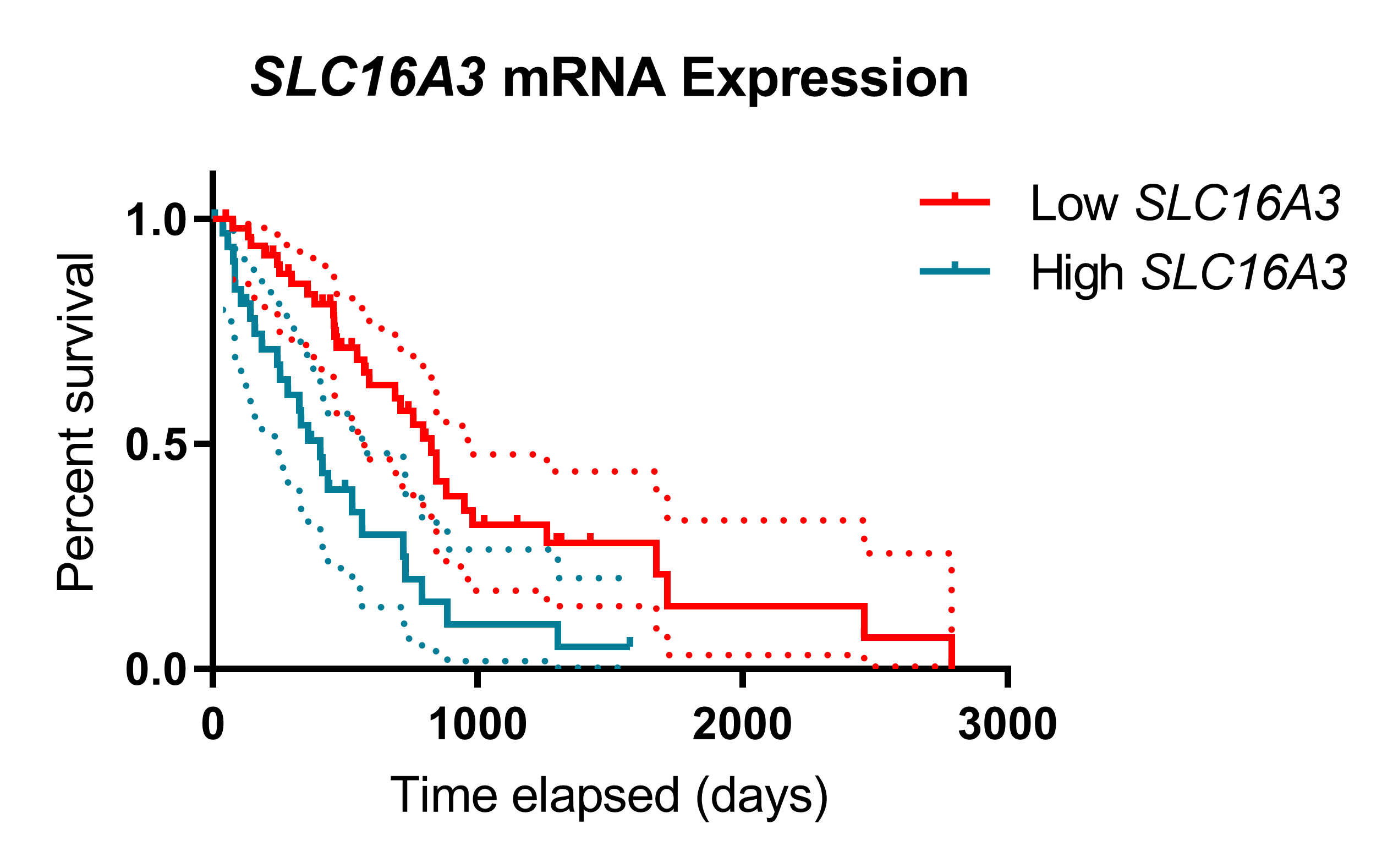

Supplement: Supplementary file 5 — Additional file 5: Figure S4. Kaplan-Meier analysis of the association of SLC16A3 (encoding for MCT4) expression with OS for patients recorded in the Mesothelioma TCGA database (“TCGA-MESO”). Blue line, patients showing high (H) marker expression; red line, patients showing low (L) marker expression. [file 12967_2020_2487_MOESM5_ESM.tif]

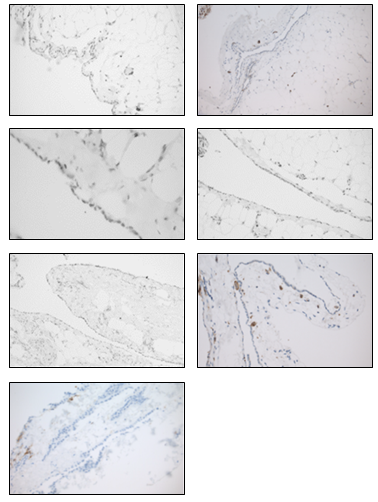

Supplement: Supplementary file 6 — Additional file 6: Figure S5. MCT4 staining in normal pleura samples. Seven representative pictures of fifteen normal pleura samples (acquired at 200X) showing that the staining with the MCT4 antibody in normal mesothelial cells provides largely negative results. [file 12967_2020_2487_MOESM6_ESM.tif]

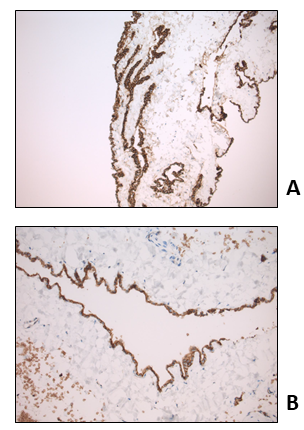

Supplement: Supplementary file 7 — Additional file 7: Figure S6. Basigin and MCT1 staining in normal pleura samples. Representative pictures of normal pleura samples showing that the staining with (A) basigin (acquired at 200X) or (B) MCT1 antibody (acquired at 200X) in normal mesothelial cells provides largely positive results. [file 12967_2020_2487_MOESM7_ESM.tif]
