# Supplementary material for: Tissue expression of lactate transporters (MCT1 and MCT4) and prognosis of malignant pleural mesothelioma (brief report)
Source: J Transl Med. 2020 Sep 4;18:341. doi: 10.1186/s12967-020-02487-6 (PMC7650278; doi:10.1186/s12967-020-02487-6)
Supplement: Supplementary file 2 — Additional file 2: Table S1. Correlation between IHC staining of BSG, MCT1, and MCT4, expressed as Pearson’s correlation coefficient r, and 95% confidence interval (95%Cl), r2 and P-value of the simple regression model. Statistical significance is indicated by asterisks (*), where * = P < 0.05; ** = P < 0.01; *** = P < 0.001, and **** = P < 0.0001. [file 12967_2020_2487_MOESM2_ESM.docx]

**Supp. Table 1:** Correlation between IHC staining of basigin, MCT1, and MCT4, expressed as Pearson's correlation coefficient r, and 95% confidence interval (95%Cl), r^2^ and P-value of the simple regression model.

|  | MCT1 vs MCT4 | MCT1 vs BSG | MCT4 vs BSG |
| --- | --- | --- | --- |
| Pearson-r | 0,039 | 0,82 | 0.069 |
| 95%Cl | -0.21-0.28 | 0.73-0.88 | -0.18-0.31 |
| r^2^ | 0.0015 | 0.67 | 0.0048 |
| P-regression | 0.76 | <0.0001^****^ | 0.58 |
