# Supplementary material for: Tissue expression of lactate transporters (MCT1 and MCT4) and prognosis of malignant pleural mesothelioma (brief report)
Source: J Transl Med. 2020 Sep 4;18:341. doi: 10.1186/s12967-020-02487-6 (PMC7650278; doi:10.1186/s12967-020-02487-6)
Supplement: Supplementary file 8 — Additional file 8: Additional literature references. Extensive literature references reporting MCT4 as putative biomarker in human cancers. [file 12967_2020_2487_MOESM8_ESM.docx]

**Additional references**

**BREAST CANCER:**

- J Doyen, C Trastour, F Ettore, I Peyrottes, N Toussant, J Gal, K Ilc, D Roux, S K Parks, J M Ferrero and J Pouysségur (2014). Expression of the hypoxia-inducible monocarboxylate transporter MCT4 is increased in triple negative breast cancer and correlates independently with clinical outcome. *Biochem Biophys Res Commun*, 451 (1), 54-61
- Yuanyuan Zhai, Lijun Chai and Jianzhong Chen (2017). The Relationship Between the Expressions of Tumor Associated Fibroblasts Cav-1 and MCT4 and the Prognosis of Papillary Carcinoma of Breast. *Pak J Pharm Sci,* 30 (1 Suppl), 263-272.
- Maria Cláudia de B Luz, Matheus M Perez, Ligia A Azzalis, Luiz Vinícius de A Sousa, Fernando Adami, Fernando L A Fonseca and Beatriz da C A Alves (2017). Evaluation of MCT1, MCT4 and CD147 Genes in Peripheral Blood Cells of Breast Cancer Patients and Their Potential Use as Diagnostic and Prognostic Markers. *Int J Mol Sci*, 18 (4).

**UROTHELIAL CARCINOMA OF THE BLADDER (UCB):**

- Jung-Woo Choi, Younghye Kim, Ju-Han Lee and Young-Sik Kim (2014). Prognostic Significance of Lactate/Proton Symporters MCT1, MCT4, and Their Chaperone CD147 Expressions in Urothelial Carcinoma of the Bladder. *Urology*, 84 (1), 245.e9-15.

**PANCREATIC DUCTAL ADENOCARCINOMA (PDA):**

- GuemHee Baek, Yan F Tse, Zeping Hu, Derek Cox, Noah Buboltz, Peter McCue, Charles J Yeo, Michael A White, Ralph J DeBerardinis, Erik S Knudsen and Agnieszka K Witkiewicz (2014). MCT4 Defines a Glycolytic Subtype of Pancreatic Cancer With Poor Prognosis and Unique Metabolic Dependencies. *Cell Rep,* 9 (6), 2233-49.
- Jack Hutcheson, Uthra Balaji, Matthew R Porembka, Megan B Wachsmann, Peter A McCue, Erik S Knudsen and Agnieszka K Witkiewicz (2016). Immunologic and Metabolic Features of Pancreatic Ductal Adenocarcinoma Define Prognostic Subtypes of Disease. *Clin Cancer Res,* 22 (14), 3606-17.
- Erik S Knudsen, Uthra Balaji, Elizaveta Freinkman, Peter McCue and Agnieszka K Witkiewicz (2016). Unique Metabolic Features of Pancreatic Cancer Stroma: Relevance to the Tumor Compartment, Prognosis, and Invasive Potential. *Oncotarget*, 7 (48), 78396-78411.

**HEPATOCELLULAR CARCINOMA (HCC):**

- Heng-Jun Gao, Ming-Chi Zhao, Yao-Jun Zhang, Dong-Sheng Zhou, Li Xu, Guang-Bing Li, Min-Shan Chen and Jun Liu (2015). Monocarboxylate Transporter 4 Predicts Poor Prognosis in Hepatocellular Carcinoma and Is Associated With Cell Proliferation and Migration. *J Cancer Res Clin Oncol,* 141 (7), 1151-62.
- Hai-Long Chen, Han-Yue OuYang, Yong Le, Peng Jiang, Hui Tang, Zi-Shan Yu, Min-Ke He, Yun-Qiang Tang and Ming Shi (2018). Aberrant MCT4 and GLUT1 Expression Is Correlated With Early Recurrence and Poor Prognosis of Hepatocellular Carcinoma After Hepatectomy. *Cancer Med*, 7 (11), 5339-5350

**GASTRIC CANCER:**

- Z Zhao, F Han, Y He, S Yang, L Hua, J Wu and W Zhan (2014). Stromal-epithelial Metabolic Coupling in Gastric Cancer: Stromal MCT4 and Mitochondrial TOMM20 as Poor Prognostic Factors. *Eur J Surg Oncol*, 40 (10), 1361-8.
- Ji Yun Lee, InKyoung Lee, Won Jin Chang, Su Min Ahn, Sung Hee Lim, Hae Su Kim, Kwai Han Yoo, Ki Sun Jung, Haa-Na Song, Jin Hyun Cho, Sun Young Kim, Kyoung-Mee Kim, Soojin Lee, Seung Tae Kim, Se Hoon Park, Jeeyun Lee, Joon Oh Park, Young Suk Park, Ho Yeong Lim and Won Ki Kang (2016). MCT4 as a Potential Therapeutic Target for Metastatic Gastric Cancer with Peritoneal Carcinomatosis. *Oncotarget*, 7 (28), 43492-43503

**ORAL SQUAMOUS CELL CARCINOMA (OSCC):**

- Jiang Zhu, Yu-Nong Wu, Wei Zhang, Xiao-Min Zhang, Xu Ding, Huai-Qi Li, Meiyu Geng, Zuo-Quan Xie and He-Ming Wu (2014). Monocarboxylate Transporter 4 Facilitates Cell Proliferation and Migration and Is Associated With Poor Prognosis in Oral Squamous Cell Carcinoma Patients. *PLoS One*, 9 (1), e87904.
- Susana Simões-Sousa, Sara Granja, Céline Pinheiro, Daniela Fernandes, Adhemar Longatto-Filho, Ana Carolina Laus, Cira Danielle Casado Alves, J M Suárez-Peñaranda, Mario Pérez-Sayáns, Andre Lopes Carvalho, Fernando C Schmitt, Abel García-García and Fatima Baltazar (2016). Prognostic Significance of Monocarboxylate Transporter Expression in Oral Cavity Tumors. *Cell Cycle*, 15 (14), 1865-73.
- Joseph Curry, Patrick Tassone, Kurren Gill, Madalina Tuluc, Voichita BarAd, Mehri Mollaee, Diana Whitaker-Menezes, Ulrich Rodeck, Adam Luginbuhl, David Cognetti, William Keane and Ubaldo Martinez-Outschoorn (2017). Tumor Metabolism in the Microenvironment of Nodal Metastasis in Oral Squamous Cell Carcinoma. *Otolaryngol Head Neck Surg,* 157 (5), 798-807.

**CLEAR CELL RENAL CELL CARCINOMA (CCRCC):**

- Marco Gerlinger, Claudio R Santos, Bradley Spencer-Dene, Pierre Martinez, David Endesfelder, Rebecca A Burrell, Marcus Vetter, Ming Jiang, Rebecca E Saunders, Gavin Kelly, Karl Dykema, Nathalie Rioux-Leclercq, Gordon Stamp, Jean Jacques Patard, James Larkin, Michael Howell and Charles Swanton (2012). Genome-wide RNA Interference Analysis of Renal Carcinoma Survival Regulators Identifies MCT4 as a Warburg Effect Metabolic Target. *J Pathol*, 227 (2), 146-56.
- Marco Gerlinger, Claudio R Santos, Bradley Spencer-Dene, Pierre Martinez, David Endesfelder, Rebecca A Burrell, Marcus Vetter, Ming Jiang, Rebecca E Saunders, Gavin Kelly, Karl Dykema, Nathalie Rioux-Leclercq, Gordon Stamp, Jean Jacques Patard, James Larkin, Michael Howell and Charles Swanton (2012). Genome-wide RNA Interference Analysis of Renal Carcinoma Survival Regulators Identifies MCT4 as a Warburg Effect Metabolic Target. *J Pathol*, 227 (2), 146-56.
- Kayvan R Keshari, Renuka Sriram, Bertram L Koelsch, Mark Van Criekinge, David M Wilson, John Kurhanewicz and Zhen J Wang (2013). Hyperpolarized 13C-pyruvate Magnetic Resonance Reveals Rapid Lactate Export in Metastatic Renal Cell Carcinomas. *Cancer Res,* 73 (2), 529-38.
- Pascale Fisel, Viktoria Stühler, Jens Bedke, Stefan Winter, Steffen Rausch, Jörg Hennenlotter, Anne T Nies, Arnulf Stenzl, Marcus Scharpf, Falko Fend, Stephan Kruck, Matthias Schwab and Elke Schaeffeler (2015). MCT4 Surpasses the Prognostic Relevance of the Ancillary Protein CD147 in Clear Cell Renal Cell Carcinoma. *Oncotarget,* 6 (31), 30615-27.
- Younghye Kim, Jung-Woo Choi, Ju-Han Lee and Young-Sik Kim (2015). Expression of lactate/H⁺ Symporters MCT1 and MCT4 and Their Chaperone CD147 Predicts Tumor Progression in Clear Cell Renal Cell Carcinoma: Immunohistochemical and The Cancer Genome Atlas Data Analyses. *Hum Pathol*, 46 (1), 104-12.
- Yan-Wei Cao, Yong Liu, Zhen Dong, Lei Guo, En-Hao Kang, Yong-Hua Wang, Wei Zhang and Hai-Tao Niu (2018). Monocarboxylate Transporters MCT1 and MCT4 Are Independent Prognostic Biomarkers for the Survival of Patients With Clear Cell Renal Cell Carcinoma and Those Receiving Therapy Targeting Angiogenesis. *Urol Oncol*, 36 (6), 311.e15-311.e25.

**MELANOMA:**

- Jonhan Ho, Michelle Barbi de Moura, Yan Lin, Garret Vincent, Stephen Thorne, Lyn M Duncan, Lin Hui-Min, John M Kirkwood, Dorothea Becker, Bennett Van Houten and Stergios J Moschos (2012). Importance of Glycolysis and Oxidative Phosphorylation in Advanced Melanoma. *Mol Cancer*, 11, 76.
- Céline Pinheiro, Vera Miranda-Gonçalves, Adhemar Longatto-Filho, Anna L S A Vicente, Gustavo N Berardinelli, Cristovam Scapulatempo-Neto, Ricardo F A Costa, Cristiano R Viana, Rui M Reis, Fátima Baltazar and Vinicius L Vazquez (2016). The Metabolic Microenvironment of Melanomas: Prognostic Value of MCT1 and MCT4. *Cell Cycle*, 15 (11), 1462-70.

**SOFT TISSUE SARCOMAS (STSS):**

- Céline Pinheiro, Valter Penna, Filipa Morais-Santos, Lucas F Abrahão-Machado, Guilherme Ribeiro, Emílio C Curcelli, Marcus V Olivieri, Sandra Morini, Isabel Valença, Daniela Ribeiro, Fernando C Schmitt, Rui M Reis and Fátima Baltazar (2014). Characterization of Monocarboxylate Transporters (MCTs) Expression in Soft Tissue Sarcomas: Distinct Prognostic Impact of MCT1 Sub-Cellular Localization. *J Transl Med*, 12, 118.

**OSTEOSARCOMA:**

- Yannan Liu, Xuanwu Sun, Chunguang Huo, Chu Sun and Jianfeng Zhu (2019). Monocarboxylate Transporter 4 (MCT4) Overexpression Is Correlated with Poor Prognosis of Osteosarcoma. *Med Sci Monit*, 25, 4278-4284.

**NON-SMALL CELL LUNG CANCER (NSCLC):**

- Marte Eilertsen, Sigve Andersen, Samer Al-Saad, Yury Kiselev, Tom Donnem, Helge Stenvold, Ingvild Pettersen, Khalid Al-Shibli, Elin Richardsen, Lill-Tove Busund, Roy M Bremnes (2014). Monocarboxylate Transporters 1-4 in NSCLC: MCT1 Is an Independent Prognostic Marker for Survival. *PLoS One*, 9 (9), e105038.

**COLORECTAL CANCER:**

- Céline Pinheiro, Adhemar Longatto-Filho, Cristovam Scapulatempo, Luísa Ferreira, Sandra Martins, Luc Pellerin, Mesquita Rodrigues, Venancio A F Alves, Fernando Schmitt and Fátima Baltazar (2008). Increased Expression of Monocarboxylate Transporters 1, 2, and 4 in Colorectal Carcinomas. *Virchows Arch*, 452 (2), 139-46.
- Yoshifumi Nakayama, Takayuki Torigoe, Yuzuru Inoue, Noritaka Minagawa, Hiroto Izumi, Kimitoshi Kohno and Koji Yamaguchi (2012). Prognostic Significance of Monocarboxylate Transporter 4 Expression in Patients with Colorectal Cancer. *Exp Ther Med*, 3 (1), 25-30.
- Yukito Gotanda, Yoshito Akagi, Akihiko Kawahara, Tetsushi Kinugasa, Takefumi Yoshida, Yasuhiko Ryu, Ichitaro Shiratsuchi, Masayoshi Kage and Kazuo Shirouzu (2013). Expression of Monocarboxylate Transporter (MCT)-4 in Colorectal Cancer and Its Role: MCT4 Contributes to the Growth of Colorectal Cancer With Vascular Endothelial Growth Factor. *Anticancer Res*, 33 (7), 2941-7.
- Sandra Fernandes Martins, Ricardo Amorim, Marta Viana-Pereira, Céline Pinheiro, Ricardo Filipe Alves Costa, Patrícia Silva, Carla Couto, Sara Alves, Sara Fernandes, Sónia Vilaça, Joaquim Falcão, Herlander Marques, Fernando Pardal, Mesquita Rodrigues, Ana Preto, Rui Manuel Reis, Adhemar Longatto-Filho and Fátima Baltazar (2016). Significance of Glycolytic Metabolism-Related Protein Expression in Colorectal Cancer, Lymph Node and Hepatic Metastasis. *BMC Cancer*, 16, 535.
- Hee Kyung Kim, InKyoung Lee, Heejin Bang, Hee Cheol Kim, Woo Yong Lee, Seong Hyeon Yun, Jeeyun Lee, Su Jin Lee, Young Suk Park, Kyoung-Mee Kim and Won Ki Kang (2018). MCT4 Expression Is a Potential Therapeutic Target in Colorectal Cancer With Peritoneal Carcinomatosis. *Mol Cancer Ther*, 17 (4), 838-848.
- Yukio Abe, Yoshifumi Nakayama, Takefumi Katsuki, Yuzuru Inoue, Noritaka Minagawa, Takayuki Torigoe, Aiichiro Higure, Tatsuhiko Sako, Naoki Nagata and Keiji Hirata (2019). The Prognostic Significance of the Expression of Monocarboxylate Transporter 4 in Patients With Right- Or Left-Sided Colorectal Cancer. *Asia Pac J Clin Oncol*, 15 (2), e49-e55.

**ESOPHAGEAL ADENOCARCINOMA:**

- Heikki Huhta, Olli Helminen, Sami Palomäki, Joonas H Kauppila, Juha Saarnio, Petri P Lehenkari and Tuomo J Karttunen (2017). Intratumoral Lactate Metabolism in Barrett's Esophagus and Adenocarcinoma. *Oncotarget,* 8 (14), 22894-22902.
